# Supplementary material for: The Role of Hedgehog Signaling in the Melanoma Tumor Bone Microenvironment
Source: Int J Mol Sci. 2023 May 16;24(10):8862. doi: 10.3390/ijms24108862 (PMC10218521; doi:10.3390/ijms24108862)
Supplement: Supplementary file 1 [file ijms-24-08862-s001.zip › ijms-2334756-supplementary.pdf]

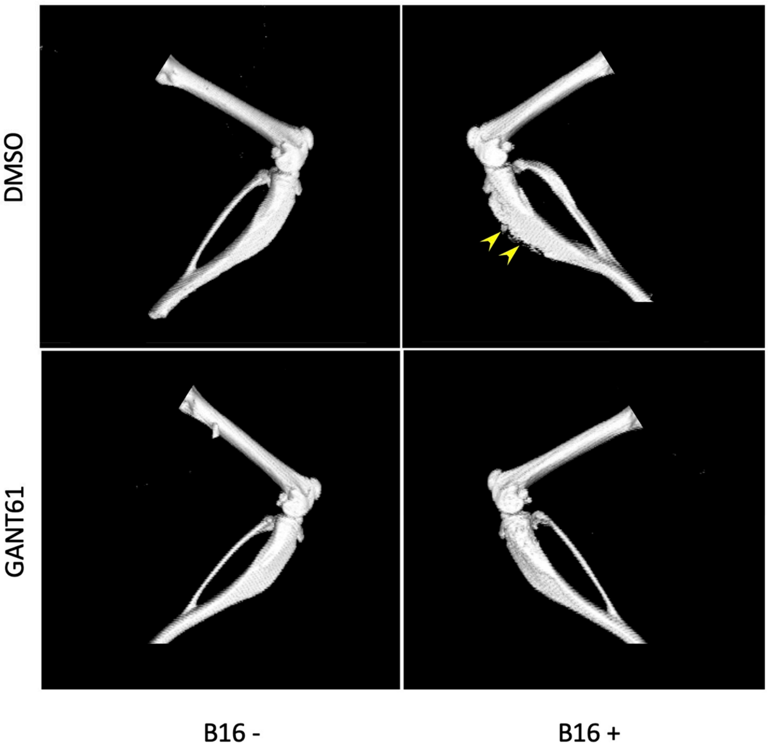

Figure S1: Computed tomography (CT) analysis of tibial metaphysis in the mouse model of bone invasion by murine B16 melanoma cells

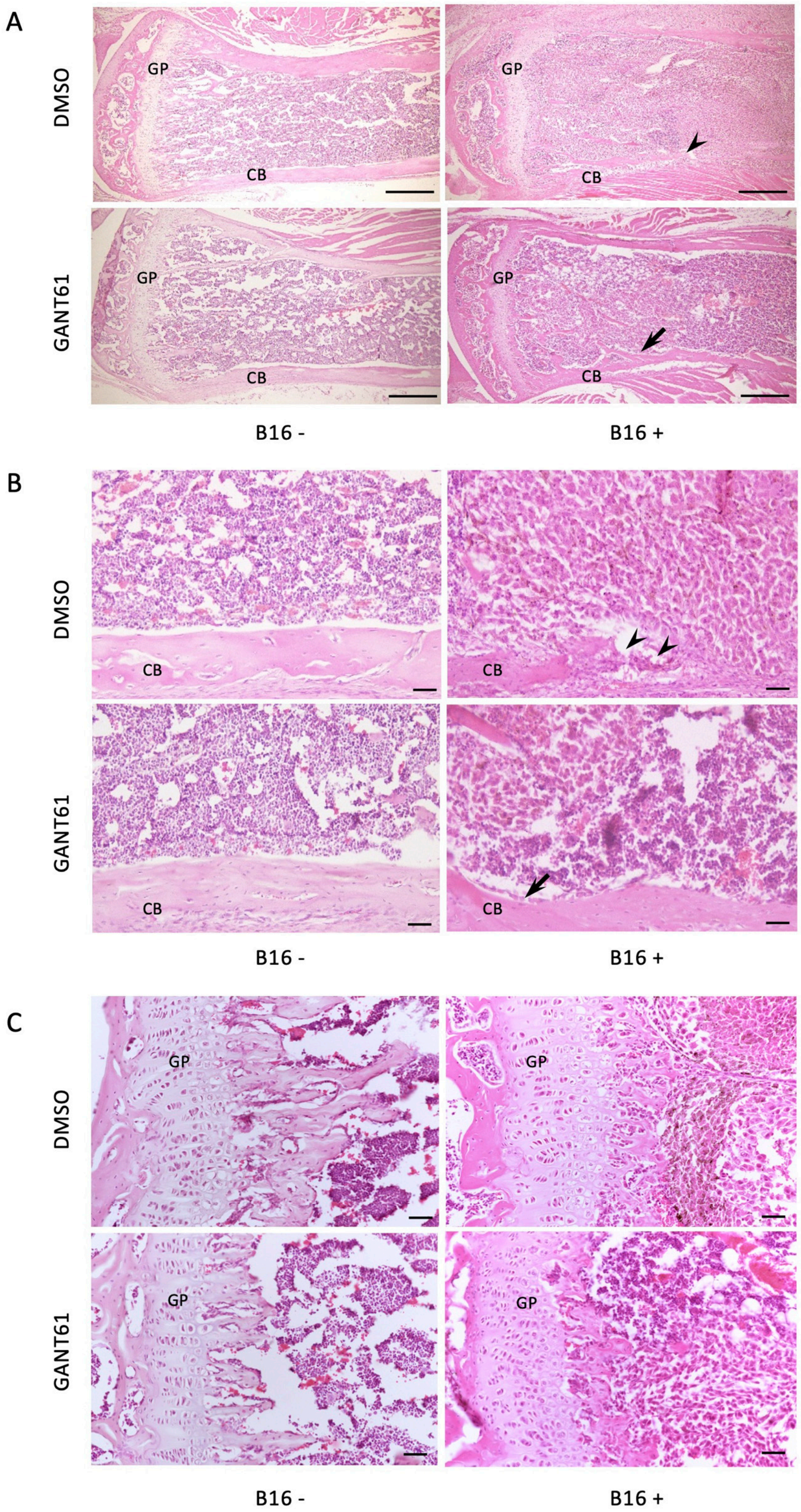

Figure S2: Hematoxylin-eosin (HE) staining of tibial metaphyses bearing B16 cells (**A**–**C**).

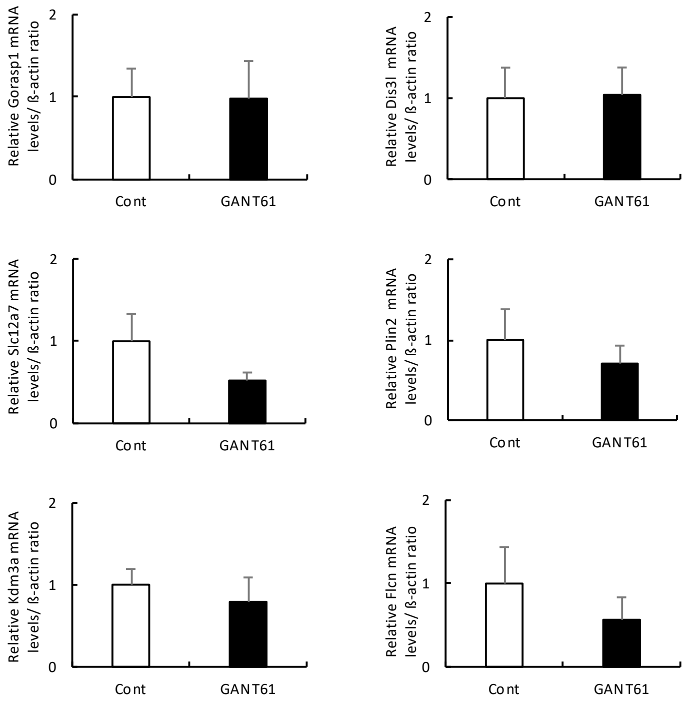

Figure S3: Expression changes in the 6 genes that were not significantly down-regulated by the exposure to GANT61

Table S1. The real-time polymerase chain reaction (PCR) primer sequences used in this study

Gene	Forward	Reverse
Tspan10	CTCTGGGGCCTGACTGTTAA	AAAGCACCCAGACATCCAGA
Car6	GCCCTCCATGTACCTTGAAA	CCCATCAATGGTGTGTTCAG
Eogt	AGGCTCAGAGGCATCCACTA	GGTGTTCTCCCAGAGTTGGA
Atp13a2	GATACTGTGGAGCGGGAACT	GGTTGTCCCCTGTCACCATA
Gorasp1	AGTCTGGGGTGTGGTATTGG	TTGTGAGGTCGTAGCTGGAG
Dis3l	TGCATGATCCTAGCCAACCA	CTTTAGCACATTCCCGGAGC
Slc12a7	GAGGTGGTGGAGATGGTTGA	CTGTCATGAATCAGCTGGGC
Plin2	GCAACTATGAACGGCTGGAG	ATCAGGTGGACAGTGGAGTG
Kdm3a	GGTATCAGAGGAGCAGGGTC	GTACAATAGCCCAGCCTTGC
Flcn	TCCTTTCTCTCAGCCTGTGG	CCAAGCCAACATACGGAAGG
β-actin	GATTACTGCTCTGGCTCCTAGC	GACTCATCGTACTCCTGCTTGC
GAPDH	AGAACATCATCCCTGCATCC	CACATTGGGGGTAGGAACAC
